# Exploring the Viral Channel Kcv_PBCV-1_ Function via Computation

**DOI:** 10.1007/s00232-018-0022-2

**Published:** 2018-02-23

**Authors:** Alma E. V. Andersson, Marina A. Kasimova, Lucie Delemotte

**Affiliations:** Science for Life Laboratory, Department of Applied Physics, KTH Royal Institute of Technology, Box 1031, SE-171 21 Solna, Sweden

**Keywords:** Viral ion channel, Homology modeling, Molecular dynamics simulations, Gating, Conduction, Protein–lipid interaction

## Abstract

Viral potassium channels (Kcv) are homologous to the pore module of complex $\hbox {K}^+$-selective ion channels of cellular organisms. Due to their relative simplicity, they have attracted interest towards understanding the principles of $\hbox {K}^+$ conduction and channel gating. In this work, we construct a homology model of the ${\hbox {Kcv}}_{\text{PBCV-1}}$ open state, which we validate by studying the binding of known blockers and by monitoring ion conduction through the channel. Molecular dynamics simulations of this model reveal that the re-orientation of selectivity filter carbonyl groups coincides with the transport of potassium ions, suggesting a possible mechanism for fast gating. In addition, we show that the voltage sensitivity of this mechanism can originate from the relocation of potassium ions inside the selectivity filter. We also explore the interaction of ${\hbox {Kcv}}_{\text{PBCV-1}}$ with the surrounding bilayer and observe the binding of lipids in the area between two adjacent subunits. The model is available to the scientific community to further explore the structure/function relationship of Kcv channels.

**Electronic supplementary material:**

The online version of this article (doi:10.1007/s00232-018-0022-2) contains supplementary material, which is available to authorized users.

## Introduction

Viral ion channels (Plugge et al. 2000) have been identified in three species of the Chlorovirus genus, which infect Chlorella-like green algae. These species are referred to as NC64A, SAG, and Pbi based on the algae organism which they infect—Chlorella NC64A, Chlorella SAG, and Chlorella Pbi, respectively.

${\hbox {Kcv}}_{\text{PBCV-1}},$ the first viral channel to be discovered, was identified in the Chlorovirus PBCV-1. This protein, while having as few as 94 amino acids per subunit, represents a fully functional potassium-selective ion channel: it is able to selectively transport potassium ions across the cell membrane along the chemical gradient. After this discovery, viral potassium channels became a focus of interest for the scientific community as the simplest protein modules functioning as potassium-selective channels. As a consequence of their relative simplicity, viral ion channels have been shown to serve as convenient building blocks when constructing potassium-selective channels with specific properties such as sensitivity to $\hbox {Ca}^{2+}$ (DiFrancesco et al. 2015), voltage (Arrigoni et al. 2013) and light (Cosentino et al. 2015).

The sequence homology between ${\hbox {Kcv}}_{\text{PBCV-1}}$ and the $\hbox {K}^+$-selective channels of other organisms allows one to infer its architecture. This protein folds into a tetrameric assembly; every subunit of this tetramer consists of transmembrane segments TM1 and TM2, an extracellular loop connecting them, and a short N-terminus. The TM1 segments build the outer surface of ${\hbox {Kcv}}_{\text{PBCV-1}}$ and face the membrane lipids, while the tetrameric assembly of TM2 defines the inner surface and lines a pathway for permeating potassium ions. The extracellular loop consists of a short helical segment, called the pore helix, and a segment lacking a secondary structure. The latter delineates the selectivity filter, a short evolutionary conserved sequence of amino acids whose role is to discriminate in favor of potassium against all other ions. In homologous potassium-selective channels, carbonyl groups of the selectivity filter residues define five ionic binding sites, called S1 (the outermost binding site) to S5 (the innermost one). Each of these sites is located between the planes defined by the carbonyl groups of two consecutive residues and provide an optimal coordination for a potassium ion. Finally, the N-terminus, whose homology to the potassium-selective channels of other organisms is the lowest, was suggested to adopt an alpha-helical conformation. Due to its amphipathic nature, this helix was thought to locate at the interface between the membrane and the intracellular solution, and was accordingly called the “slide helix” (Moroni et al. 2002; Gazzarrini et al. 2002; Hertel et al. 2010).

Expression of ${\hbox {Kcv}}_{\text{PBCV-1}}$ in Xenopus oocytes revealed that this channel has two gating modalities, slow and fast, both sensitive to changes in the transmembrane potential (Abenavoli et al. 2009). The slow modality was suggested to involve the TM1 segments: the movement of TM1 is coupled to that of the adjacent domains of the pore through salt bridges, and upon disruption of the salt bridges, TM1 interferes with the transport of potassium ions (Hertel et al. 2010; Gebhardt et al. 2011). In this case, the voltage sensitivity may result from the formation/breaking of the salt bridges. For the fast modality, which is prominent at extreme positive and negative voltages, the selectivity filter was proposed to play a crucial role (Abenavoli et al. 2009). While in ${\hbox {Kcv}}_{\text{PBCV-1}}$ the origin of the voltage sensitivity of the gating mechanism remains to be investigated, in the potassium-selective channel K2P, the movement of ions inside the selectivity filter upon channel opening was shown to carry charge, and was thus pinpointed as the origin of voltage sensitivity (Schewe et al. 2016).

In the absence of a high-resolution structure, homology modeling can be applied to build an atomistic model of a query protein. For ${\hbox {Kcv}}_{\text{PBCV-1}}$ specifically, this method was shown to provide important insights into the role of specific residues and even domains (Tayefeh et al. 2009). For example, using a ${\hbox {Kcv}}_{\text{PBCV-1}}$ homology model, Hoffgaard et al. were able to rationalize the effect of N-terminus mutagenesis on ${\hbox {Kcv}}_{\text{PBCV-1}}$ ionic conductance. The authors proposed that, in the wild type, the N-terminus forms a salt bridge with the C-terminal carboxyl group. Upon mutagenesis of the N-terminus and thus weakening of the corresponding salt bridge, the increased negative surface charge at the C-terminus causes a decrease in ionic conductance through the redistribution of cations bound inside the pore (Hoffgaard et al. 2015). In another study, the protonation state of the evolutionary conserved K29 has been assessed: based on the ${\hbox {Kcv}}_{\text{PBCV-1}}$ homology model, Gebhardt et al. suggested that K29 faces the membrane lipids and hence should be deprotonated. In agreement with their conclusion, mutagenesis of K29 to alanine was indeed tolerated by ${\hbox {Kcv}}_{\text{PBCV-1}}$ (Gebhardt et al. 2012). Finally, for the related viral ion channel Kcv-ATCV1, the homology modeling provided insight into the role of hydrogen bonds between the intracellular parts of M1 and M2 in the stabilization of the open state (Rauh et al. 2017).

In this study, we resort to homology modeling to explore the molecular origin of the ${\hbox {Kcv}}_{\text{PBCV-1}}$ fast gating and its sensitivity to voltage. Our homology model, which corresponds to the open state of ${\hbox {Kcv}}_{\text{PBCV-1}},$ is based on a high-resolution structure of a potassium-selective NaK mutant (PDB ID: 3TET (Sauer et al. 2011)) and is stable for over 300 ns of molecular dynamics simulations. To access the quality of this model, first, we confront docking of ${\hbox {Kcv}}_{\text{PBCV-1}}$ blockers with previously reported data, and second, we test whether our homology model is conductive upon application of an electric field.

We then suggest that fast gating involves conformational rearrangements of the V64, G65, and F66 residues of the selectivity filter, which seem to be affected by ions moving in this compartment. Further, to assess voltage sensitivity of fast gating, we quantify the amount of charge transferred by an ion passing through the selectivity filter. Finally, we also explore the interaction of ${\hbox {Kcv}}_{\text{PBCV-1}}$ with the membrane and, in particular, characterize lipid binding to protein residues.

## Methods

### Homology Modeling

To find a template for the homology modeling, we passed the ${\hbox {Kcv}}_{\text{PBCV-1}}$ sequence through a BLASTp search (Altschul et al. 1990). The Y66F mutant of the NaK channel (NaK2K, PDB 3TET) was identified as the protein with the highest homology to ${\hbox {Kcv}}_{\text{PBCV-1}}$ and hence was used to build a model (Sauer et al. 2011; Fassler 2011). The identity score between ${\hbox {Kcv}}_{\text{PBCV-1}}$ and NaK2K was estimated to be 53%.

**Fig. 1:**
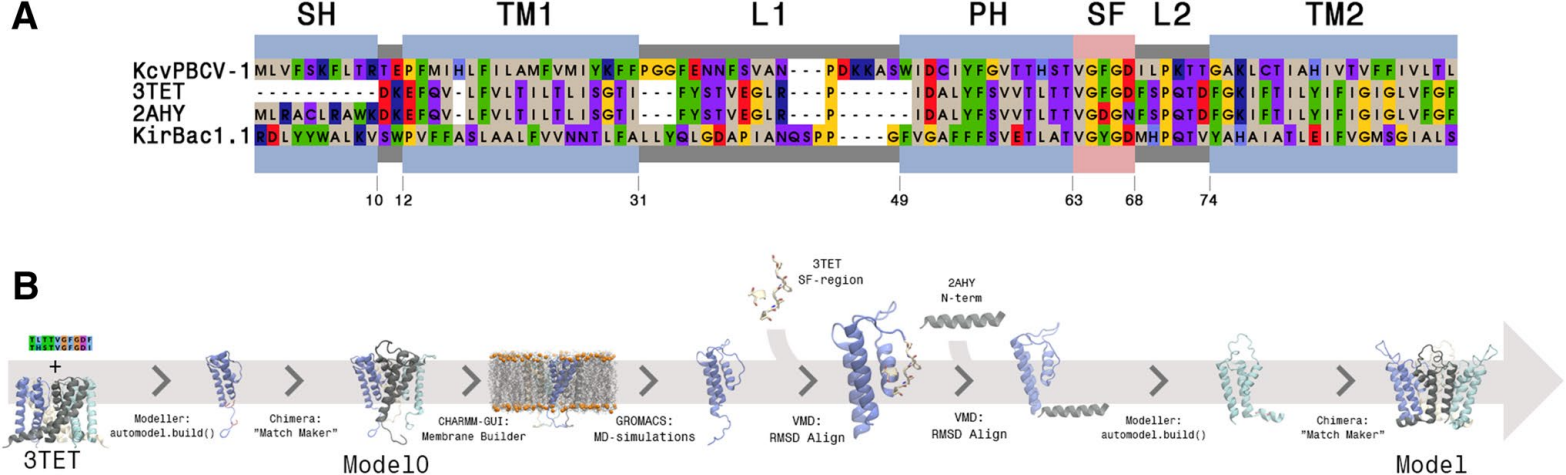
Homology modeling. **a** Sequence alignment between ${\hbox {Kcv}}_{\text{PBCV-1}}$ , the wild-type (2AHY) and potassium-selective mutant (3TET) of the NaK channel, and the KirBac1.1 template used to build an earlier model (Tayefeh et al. 2009). **b** Overview of the homology modeling protocol. The various modeling steps, starting from the template and ending with the final open ${\hbox {Kcv}}_{\text{PBCV-1}}$ model, used for molecular dynamics simulations, are shown

An overview of the homology modeling workflow is shown in Fig. 1b. The homology modeling was performed using Modeller v.9.17 (Šali and Blundell 1993). The sequences of ${\hbox {Kcv}}_{\text{PBCV-1}}$ and the Y66F mutant of NaK were aligned using the Modeller built-in alignment function. The following restraints were applied during the homology modeling procedure: $\alpha$-helical restraints on the region including residues 13–35 (TM1), predicted to be a transmembrane helix by the TMHMM web-service (Moller et al. 2001); and Gaussian distance restraints on the Y55-D68 pair, previously determined to be essential for the selectivity of potassium channels (Sauer et al. 2011). A total of 10 different models of the ${\hbox {Kcv}}_{\text{PBCV-1}}$ monomeric unit were generated. Models with a selectivity filter (residues 60 to 68) deviating substantially from the template were discarded. Out of those, the model with the lowest DOPE-score (Shen and Sali 2006) was used to construct a tetrameric assembly. In practice, the bioassembly structure of NaK2K was used as a reference: the selectivity filter region (TVGFG) of each monomeric unit was aligned to that of the reference using the Match Maker tool in UCFS Chimera v.1.11.2. (Pettersen et al. 2004). This procedure yielded Model 0.

Model 0 was then inserted into a membrane/solution environment and molecular dynamics simulations were performed (as described below). The simulations revealed that the transmembrane part of this model was stable, while the N-terminus deviated substantially from the initial conformation and between different subunits. Therefore, we performed another homology modeling step, in which we applied additional restraints on the N-terminus. As a template for residues 13–59, 61–62, and 69–94, we used a monomer of the equilibrated Model 0; for residues 60 and 63-68 of the selectivity filter—the Y66F mutant of NaK; and for residues 1–10 of the N-term—the WT NaK channel (PDB 2AHY) (Fig. 1) (Shi et al. 2006). Model 0 and the WT NaK channel were structurally aligned on the TM1 segments, and Model 0 and NaK2K—on the selectivity filter. Tetramerization was performed using the same procedure as for Model 0. This procedure yielded the final model further described in the results and discussion section.

### System Preparation

The two models (Model 0 and the final model) were embedded in a palmitoyl-oleoyl-phosphatidylcholine (POPC) bilayer using the CHARMM-GUI Membrane Builder (Jo et al. 2008). The following configuration of the ${\hbox {Kcv}}_{\text{PBCV-1}}$ selectivity filter was considered: three potassium ions in the S2, S4, and S5 binding sites and two water molecules in the S1 and S3 binding sites—{W,S2,W,S4,S5}. The system was further hydrated: two water layers of 25.0 Å thickness were added to both sides of the membrane. K$^+$ and Cl$^-$ ions were added using the Monte-Carlo method to yield a concentration of 150 mM.

### Molecular Dynamics (MD) Simulations

MD simulations were performed using GROMACS v. 2016.3 (Berendsen et al. 1995; Lindahl et al. 2001; Pronk et al. 2013a, b; Abraham et al. 2015). The CHARMM36 force field (Best et al. 2012; Klauda et al. 2010) was used to model the interactions within the protein and the lipid bilayer, and the TIP3P model—to describe water molecules (Jorgensen et al. 1983). The protocol used for the two systems is shown in Table 1. The short-range electrostatic interactions were modeled with a 1.2-nm cutoff, considering a switching function on the interval between 1.0 and 1.2 nm. The long-range electrostatic interactions were computed using Particle Mesh Ewald (PME) (Darden et al. 1993). Bonds involving hydrogens were constrained using the Linear Constraint Solver (LINCS) (Hess 2008).

In one of the simulations, a constant electric field was applied along the normal to the lipid bilayer to generate a transmembrane potential of + 500 mV. This was done by adding an external force to all charged particles in the system, as implemented in GROMACS. The trajectory for Model 0 was a total of 115 ns long, the one for the final model under no external potential, 369 ns and the one under +500 mV, 344 ns.

**Table 1:** Parameters for the MD simulations

Step	Duration (ps)	Time step (fs)	Thermostat/barostat	Backbone Force constant (kJ/mol/nm^2^)	Side chain Force constant (kJ/mol/nm^2^)
1	25	1	Berendsen/-	4000.0	2000.0
2	25	1	Berendsen/-	2000.0	1000.0
3	25	1	Berendsen/Berendsen	1000.0	500.0
4	100	2	Berendsen/Berendsen	500.0	200.0
5	100	2	Berendsen/Berendsen	200.0	50.0
6	100	2	Berendsen/Berendsen	50.0	0.0
7	1000	2	Nose-Hoover/Parrinello Rahman	0.0	0.0

### Analysis of the MD Trajectories

#### Contact Map

A contact map between the protein residues was generated using the GROMACS mdmat tool, representing the shortest distance between any two atoms of residue pairs averaged over the trajectory. The average was computed over a total of 370 equidistant frames extracted from the trajectory of the final model under no external potential. All settings were set to default, including the cutoff (truncation) of 1.5 nm.

#### Binding Site Occupancy

The occupancy of the selectivity filter binding sites was estimated using MDTraj (McGibbon et al. 2015). The occupancy of a binding site $f_i$ corresponds to the number of ions throughout the trajectory falling within a volume $V_{BS_i}$ between eight coordinating oxygens, which define the binding site, normalized by the total number of frames analyzed:

$$\begin{aligned} {f_i} = \frac{\sum _{k=0}^N w_{k,i}}{N}, \qquad w_{k,i} = {\left\{ \begin{array}{ll} 1 &{} \text {if ion } \, k \, \text { in } V_{BS_i} \\ 0 &{} \text {else} \end{array}\right. } \end{aligned}$$

#### Orientation of the Selectivity Filter Carbonyls

To characterize the orientation of the selectivity filter carbonyls, we considered two variables: (1) distance of the carbonyl oxygen to the channel’s main axis and (2) $(C, C_\alpha , N, O)$ dihedral. We estimated these two variables along the MD trajectory (Fig. 8), and clustered the data using the DBSCAN module of scikit-learn v.0.19.0. (Pedregosa et al. 2011; Ester et al. 1996). Then we assigned the clusters corresponding to inward- or outward-oriented carbonyls based on the comparison to two reference points (structures representing conductive states of potassium-selective channels: human ether-a-go-go related channel (PDB ID: 5VA1) and the Y66F mutant of NaK (PDB ID: 3TET)) (Sauer et al. 2011; Wang and MacKinnon 2017).

#### Electrical Distance Calculation

The gating charge *Q* associated with the rearrangement of the system between conformational states $\mathbf {\lambda }_1$ and $\mathbf {\lambda }_2$ can be expressed through

$$\begin{aligned} Q = \frac{{\varDelta }G(\varvec{\lambda }_2, {\varDelta }V) - {\varDelta }G(\varvec{\lambda }_1, {\varDelta }V)}{{\varDelta }V}, \end{aligned}$$

where ${\varDelta }G(\mathbf {\lambda }, {\varDelta }V)$ is the excess free energy due to the applied voltage ${\varDelta }V.$ The excess free energy relates a system state $\mathbf {\lambda }$ to the electrical distance $\delta _i^{\mathbf {\lambda }}$ (Delemotte et al. 2011; Sigworth 1994):

$$\begin{aligned} {\varDelta }G(\varvec{\lambda }, {\varDelta }V) = G(\varvec{\lambda }, {\varDelta }V) - G(\varvec{\lambda }, 0) = {\varDelta }V \times \sum _i q_i \times \delta _i^{\varvec{\lambda }}, \end{aligned}$$

where $q_i$ is the $\hbox {i}^{th}$ charge. The electrical distance at the position of the charge $q_i$ therefore reports how much charge can be transferred by $q_i$ and it can be calculated using

$$\begin{aligned} \delta _i^{\mathbf {\lambda }} = \frac{\partial {\varPhi }_i^{\mathbf {\lambda }}}{\partial V}\Bigr |_{V=0}, \end{aligned}$$

where ${\varPhi }(\mathbf {r},{\varDelta }V)$ is the local electrostatic potential at position $\mathbf {r}$ and under a potential ${\varDelta }V.$ It is obtained by solving Poisson’s equation on a grid (Aksimentiev and Schulten 2005):

$$\begin{aligned} \nabla ^2 {\varPhi }(\mathbf {r},{\varDelta }V) = -4 \pi \sum _i \rho _i(\mathbf {r}), \end{aligned}$$

where $\rho _i$ is the point charge approximated by a spherical Gaussian of inverse width $\sigma,$ and the sum runs over all atoms in the system. For our calculations, we considered a grid of 1.5 $\times$ 1.5 $\times$ 1.5 Å and $\sigma$ of 0.15 Å.

We calculated ${\varDelta }V$ as the difference between the local electrostatic potential at +500 and 0 mV normalized by the applied voltage. In practice, from the trajectory, we extracted several ${\hbox {Kcv}}_{\text{PBCV-1}}$ conformations, differing in the ionic occupancy of the selectivity filter. For each of them, we generated two 5 ns trajectories, one under + 500 mV and the other under 0 mV. From these, we considered 250 frames extracted every 20 ps to calculate ${\varPhi }(\mathbf {r},{\varDelta }V).$

#### Interactions with a Lipid Bilayer

The average occupancy of the lipids was calculated using the VolMap tool of VMD (Humphrey et al. 1996). For the analysis, we extracted one frame every 1 ns of the trajectory and averaged lipid position over these. In addition to the average occupancy, for each ${\hbox {Kcv}}_{\text{PBCV-1}}$ residue, we also calculated the number of lipid atoms within the radial cutoff distance of 1 Å of any residue atom.

### Docking

Docking of five binders to ${\hbox {Kcv}}_{\text{PBCV-1}}$ was carried out using AutoDock Vina (Trott and Olson 2010). The final model was used as a receptor, whilst five compounds (Bretylium Tosylate, Amantadine, Rimantadine, Sotalol, and TEA) were used as ligands. The binding equilibrium constant $K_i$ of these compounds was previously determined experimentally (Tan et al. 2012; Plugge et al. 2000; Syeda et al. 2008; Chatelain et al. 2009). Structures of all ligands were downloaded in a PDB-format from the DrugBank v.5.0 database (Law et al. 2014; Knox et al. 2011; Wishart et al. 2008, 2006).

The docking procedure consisted of two steps. The first grid box was set to include the entire protein. Based on the obtained results, a second grid box, including the area of the 10 best docking poses, was considered to limit the volume of search.

To compare with the experimental data, the ligand with the best docking score was chosen and the corresponding $K_i$ was estimated using the obtained binding free energy ${\varDelta }G:$

$$\begin{aligned} K_i = e^{\frac{{\varDelta }G}{RT}}, \end{aligned}$$

where $R = 8.3145 J\times K^{-1}\times mol^{-1.}$ and $T=298.15 K.$ Whilst the $K_i$ for the experimental results are given in units of micro-or millimolars, the ligand concentrations are low and the solution can be taken to be dilute, allowing the numerical value of the coefficient of activity to be set as 1. The adjusted dimensionless $K_i$ value we used for comparison with the docking results is therefore equal in magnitude to that of the experimental values but compatible with the necessary exponential transformation.

## Results and Discussion

### Homology Modeling

A homology model of ${\hbox {Kcv}}_{\text{PBCV-1}}$ had been previously built by Tayefeh et al. (2009) based on the high-resolution structure of KirBac1.1 (Kuo et al. 2003). Since then several structures of other homologous channels have been resolved, suggesting the possible availability of a template with higher sequence similarity. To test this possibility, we passed the ${\hbox {Kcv}}_{\text{PBCV-1}}$ sequence through BLASTp (Altschul et al. 1990), which is commonly used to search homologous proteins in a database (in our case, in the Protein Data Bank). This analysis has identified the $\hbox {K}^+$-selective mutant of the NaK channel (NaK2K) to share the highest identity with ${\hbox {Kcv}}_{\text{PBCV-1}}:$ the corresponding identity score was estimated to be 53% (Fig. 1a). The structure of this mutant (PDB ID: 3TET (Sauer et al. 2011)) was further used to build a ${\hbox {Kcv}}_{\text{PBCV-1}}$ homology model (see Methods section). In addition, we used the N-terminus of the wild-type NaK channel (PDB ID: 2AHY (Shi et al. 2006)) as a template for the corresponding segment in ${\hbox {Kcv}}_{\text{PBCV-1}}$ (residues 1–10), due to the fact that in the NaK mutant the N-terminus has not been resolved. The protocol of the homology modeling can be summarized in two steps (Figure 1b). First, we built a homology model (Model 0) without applying any restraints to the N-terminus. To assess the quality of this model, we tested its ability to conduct ions upon application of an electric field. Second, using Model 0, which was shown to be conductive, we built a final homology model, applying “slide helix” restraints to the N-terminus (see Methods, Figure S1). This final homology model was further used as a starting conformation for extensive molecular dynamics (MD) simulations (see Methods and Fig. 1b).

**Fig. 2:**
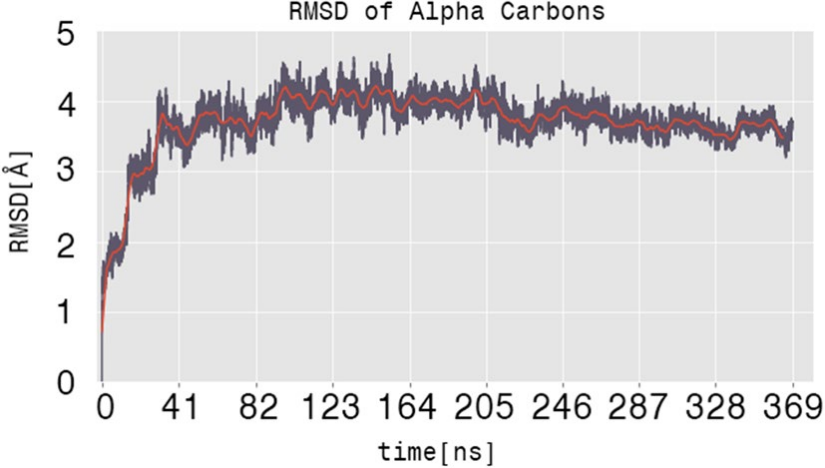
Root mean square deviation (RMSD) of all ${\hbox {Kcv}}_{\text{PBCV-1}}$ conformations sampled along the 369 ns MD simulation under no applied voltage. For the analysis, the $\hbox {C}_\alpha$ atoms of the protein were considered. The initial configuration was used as a reference

To assess the quality of the open state ${\hbox {Kcv}}_{\text{PBCV-1}}$ model, we estimated the root mean square deviation (RMSD) of all protein conformations along the MD simulations with respect to the starting structure. The RMSD is commonly used to report the stability of a model in a membrane/solution environment. Here, it reaches a plateau at ~4 Å after 100 ns (Fig. 2), suggesting that the homology model is stable during the molecular dynamics simulations (369 ns in total).

**Fig. 3:**
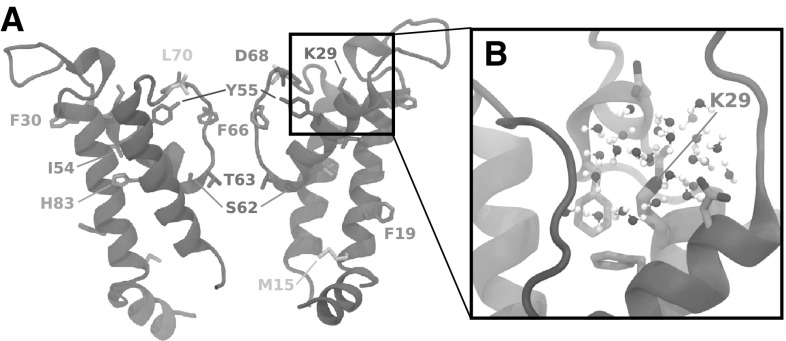
Location of the ${\hbox {Kcv}}_{\text{PBCV-1}}$ residues, whose role in the function of this channel has been previously suggested based on the mutagenesis experiments (Table 2). A. ${\hbox {Kcv}}_{\text{PBCV-1}}$ side view; two subunits (in gray) out of four are shown for clarity. The residues explored in the previous studies are represented as sticks and are shown in different colors. B. Environment of K29 in the homology model. The protein residues and the water molecules located within 5 Å of K29 are shown. Both the protein residues and the water molecules are colored by the atom name: oxygen—red, hydrogen—white, and carbon—cyan

Interestingly, in our homology model, the evolutionary conserved K29 faces the extracellular solution (Fig. 3) and not the lipid bilayer as in the model by Tayefeh et al. (2009). This difference in the position of K29 is a result of using a different template for the homology modeling (see Section Homology and Modeling). In the model by Tayefeh et al., a positively charged K29 was shown to attract excessive water inside the lipid bilayer, thus yielding an unstable model. Based on this observation and on mutagenesis experiments (Gebhardt et al. 2012), the authors suggested that in ${\hbox {Kcv}}_{\text{PBCV-1}}$ K29 is deprotonated. Here, however, we report that the deprotonation of K29 is not necessary if a template with a higher identity score is used. In addition, we report that the location of another residue, M15, agrees with known experimental data, as was the case in the previous model (Tayefeh et al. 2009): this residue was shown to be sensitive to water-soluble oxidizing agents (Schroeder et al. 2013) and, in our model, it is consistently facing the intracellular solution (Fig. 3 and Table 2).

**Table 2:** ${\hbox {Kcv}}_{\text{PBCV-1}}$ residues of functional importance, as reported previously

Residue	Functional implication	References
D68, Y55	The interaction between D68 and Y55 is important for the stability of the selectivity filter.	Sauer et al. (2011)
F19, I54, F66	These residues are involved in a network of long-distance interactions	Gazzarrini et al. (2004)
T63, S62	T63S results in the loss of ${\hbox {Kcv}}_{\text{PBCV-1}}$ sensitivity to amantadine, while S62T restores it partially	Plugge et al. (2000), Chatelain et al. (2009)
L70	Mutagenesis of L70 affects ${\hbox {Kcv}}_{\text{PBCV-1}}$ conductance at negative voltages, and sensitivity to TEA	Tan et al. (2012)
M15	Sensitive to water-soluble oxidizing agents	Schroeder et al. (2013)
K29	K29A does not abolish the channel function	Gebhardt et al. (2012)
F30, H83	Mutating these residues is not tolerated in yeast growth assays.	Gebhardt et al. (2011)

### Docking of the ${\hbox {Kcv}}_{\text{PBCV-1}}$ Blockers to the Homology Model

**Fig. 4:**
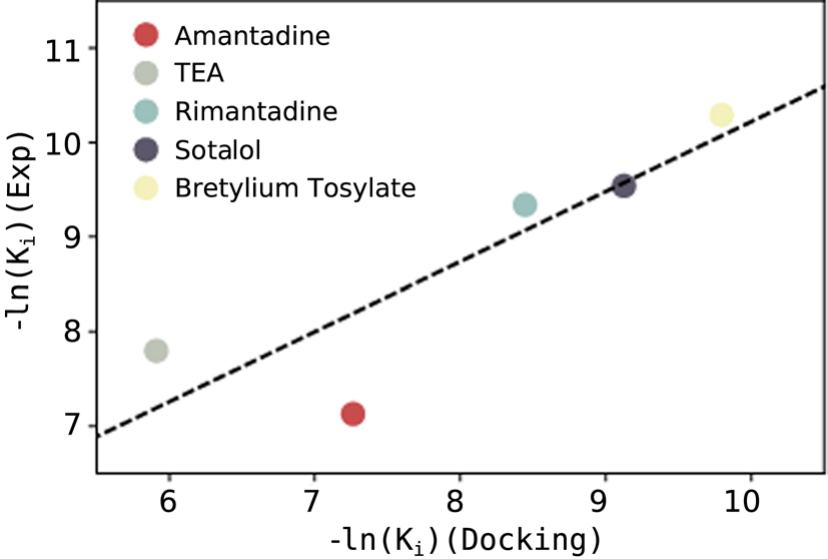
Correlation between the binding affinities predicted from docking and those estimated experimentally. The *R* value is 0.87

**Fig. 5:**
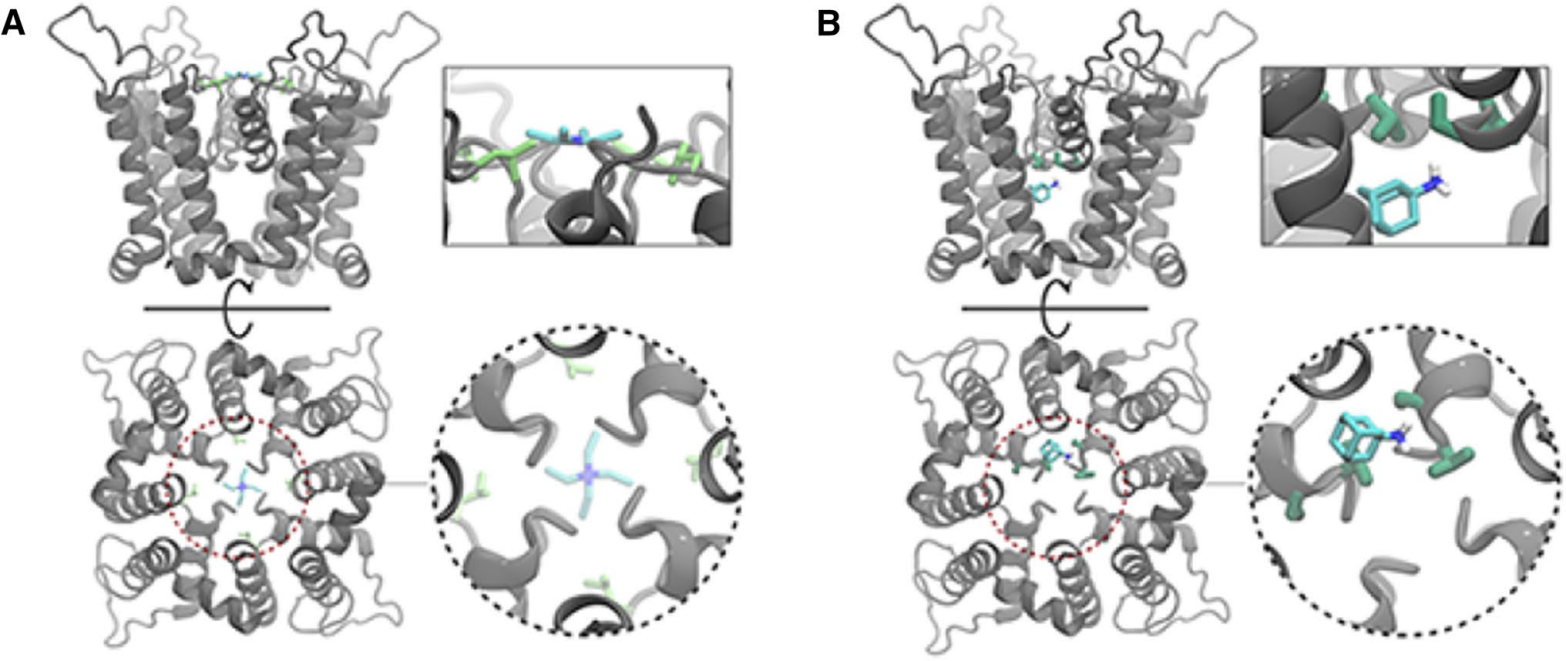
Highest ranked docking poses for TEA (**a**) and amantadine (**b**). The side (top panel) and bottom (bottom panel) views are shown. The rectangular and circular insets correspond to the zoomed-in views on the blockers. TEA and amantadine are colored by the atom name: nitrogen—blue, hydrogen—white, and carbon—cyan. L70 and T63 are shown in light-green and dark-green, respectively

To further assess the quality of our homology model, we docked several known ${\hbox {Kcv}}_{\text{PBCV-1}}$ blockers to it, for which the binding affinities and the interacting residues are known. In total, we chose five different blockers: amantadine, tetra-ethyl-ammonium (TEA), rimantadine, sotalol, and bretylium tosylate. Figure 4 shows that the binding affinities predicted from the docking simulations and those estimated experimentally correlate strongly (*R* value = 0.87), suggesting that the homology model satisfactorily describes the interactions between the ${\hbox {Kcv}}_{\text{PBCV-1}}$ and its blockers. Previous mutagenesis experiments revealed that L70 and T63 are involved in the binding of TEA and amantadine, respectively. In agreement with these data, we show that in the highest ranked docking poses, these two blockers are indeed located in the vicinity of L70 and T63 (Fig. 5). In addition, we docked the blockers to the ${\hbox {Kcv}}_{\text{PBCV-1}}$ equilibrated conformation; the resulting docking poses were shown to be similar to those obtained for the homology model (Supplementary Figs. S3, S4) Table 3.

**Table 3:** Binding affinities ($\hbox {K}_i$) estimated from the docking simulations and experimentally. Experimental $\hbox {K}_i$ values were rendered unitless considering an activity coefficient of 1

Blocker	$\hbox {K}_i$ (Docking)	$\hbox {K}_i$ (Experiments)
Amantadine	$7.04 \times 10^{-4}$	$8.0 \times 10^{-4}$
TEA	$2.71 \times 10^{-3}$	$4.1 \times 10^{-4}$
Rimantadine	$2.15 \times 10^{-4}$	$8.8 \times 10^{-5}$
Sotalol	$1.09 \times 10^{-4}$	$7.2 \times 10^{-5}$
Bretylium Tosylate	$5.57 \times 10^{-5}$	$3.4 \times 10^{-5}$

### Ionic Transport Through the Selectivity Filter Upon Application of Voltage

**Fig. 6:**
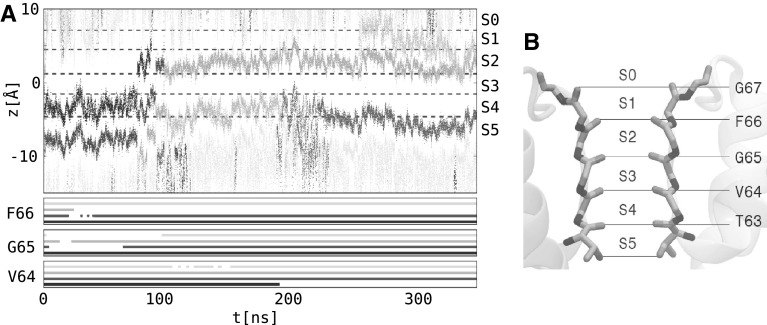
**a** Conduction events and conformational changes of the backbone of the selectivity filter. The top panel shows the position of ions along the ${\hbox {Kcv}}_{\text{PBCV-1}}$ principal axis. Ions, entering the selectivity filter, are represented in different colors; those located in the ${\hbox {Kcv}}_{\text{PBCV-1}}$ cavity and the extracellular solution are shown in light gray. The dashed lines separate the binding sites of the selectivity filter (S0–S5). The bottom panel shows the conformational changes of the backbone of V64, G65, and F66. The carbonyl groups of these residues fluctuate between two alternative states, facing either the pore (continuous lines) or the pore helices (interruptions in the continuous lines). The four lines per residue correspond to different subunits of the channel. **b** Selectivity filter binding sites: S0–S5. The backbone of the selectivity filter is shown as sticks. The binding sites S0–S5 are located between the planes, which are defined by the backbone carbonyl groups (or side chain hydroxyl in the case of T63) of the T63-G67 residues

**Fig. 7:**
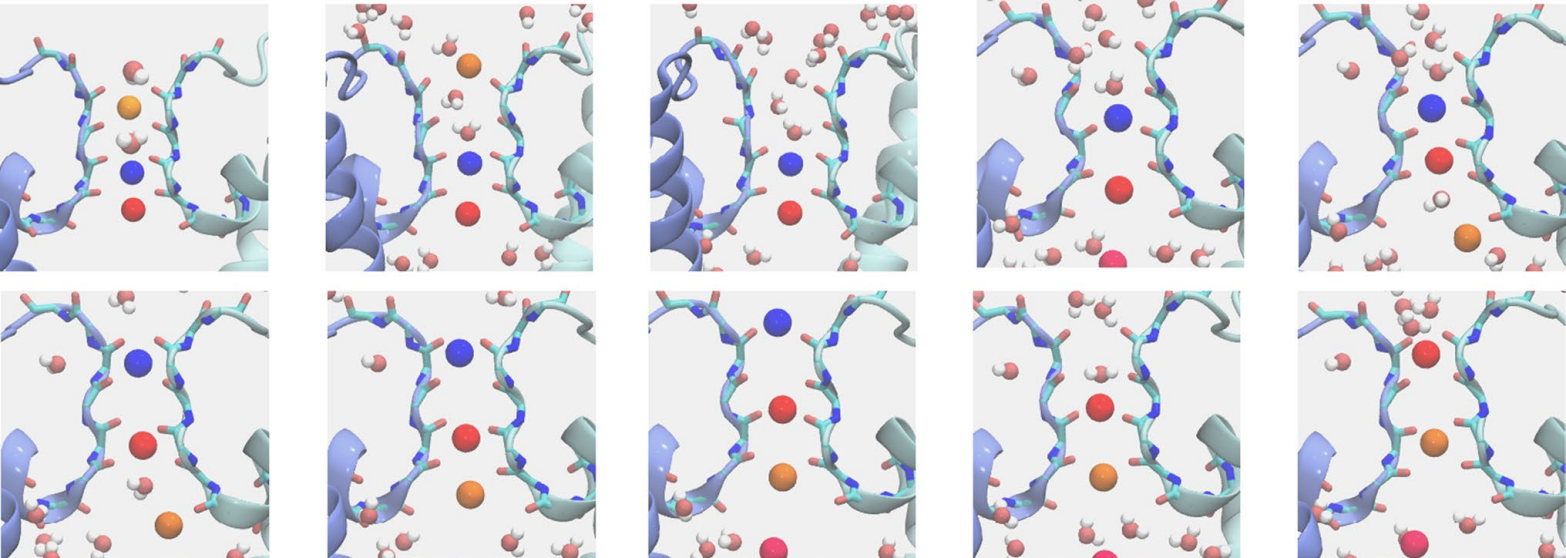
Conduction events shown in several representative snapshots. The ions passing through the selectivity filter are colored according to Fig. 6. The backbone of the selectivity filter is shown as sticks. For the details of the translocation events, see text

Since the homology model of ${\hbox {Kcv}}_{\text{PBCV-1}}$ corresponds to an open state, we tested its ability to conduct potassium ions. The previous model has demonstrated the ability to support ion rearrangement in the selectivity filter, but the short timescales of the simulations performed (50 ns) did not allow to describe full translocation events. In this work, we performed a  350 ns molecular dynamics simulation of the open model in conditions of a depolarizing transmembrane potential (+500 mV) and monitored the positions of potassium ions along the channel principal axis (Figs. 6 and 7 and Supplementary Fig. S5 for the Model 0 results).

In the starting configuration, three ions were present in the selectivity filter binding sites S2, S4, and S5 and two water molecules were occupying S1 and S3. We will refer to this configuration as {W,S2,W,S4,S5}. Shortly after the application of an electric field, we noticed that one of the ions relocates from S2 to S1 and finally leaves the channel, resulting in the configuration {W,W,W,S4,S5}. After that, the ion in S4 jumps to S3 and then to S2; as soon as S4 becomes vacant, the ion from S5 relocates to this binding site. This sequence of the configurations can be summarized as the following:

$$\begin{aligned}&\{W,W,W,S4,S5\} \rightarrow \{W,W,S3,0,S5\} \\&\quad \rightarrow \{W,W,S3,S4,W\} \rightarrow \{W,S2,0,S4,W\} \end{aligned}$$

In the next steps, the ion approaching from the ${\hbox {Kcv}}_{\text{PBCV-1}}$ intracellular cavity binds at the level of S5, pushing the other two ions inside the selectivity filter to move one step upwards:

$$\begin{aligned} \{W,S2,0,S4,S5\} \rightarrow \{S1,0,S3,0,S5\} \end{aligned}$$

Finally the ion in S1 leaves the channel, and the selectivity filter adopts the configuration that we have already observed, {W,W,S3,0,S5}, closing the cycle of a potassium ion transport. Overall, we counted two full translocation events across the selectivity filter for the presented homology model (Fig. 6, see also Figure S5 for translocation events occurring in Model 0). The inferred conductance amounts to  1 pA, which is about two orders of magnitude smaller than the measured conductance of ${\hbox {Kcv}}_{\text{PBCV-1}}$ in symmetric 100 mM KCl in Xenopus oocytes. Such a small conductance is likely a result of force fields that overly stabilize the ions in the selectivity filter of potassium channels, as reported in studies of $\hbox {K}^+$ conduction in other potassium channels (Köpfer et al. 2014).

The inferred mechanism of ionic transport at the level of the selectivity filter agrees qualitatively with a mechanism described previously using MD simulations of other $\hbox {K}^+$-selective channels (Köpfer et al. 2014). In this mechanism, referred to as “hard knock-on,” water molecules do not intercalate between potassium ions during the ionic transport. This agreement provides further confidence in the open state ${\hbox {Kcv}}_{\text{PBCV-1}}$ homology model reported here.

### A Possible Mechanism of Gating at the Selectivity Filter

**Fig. 8:**
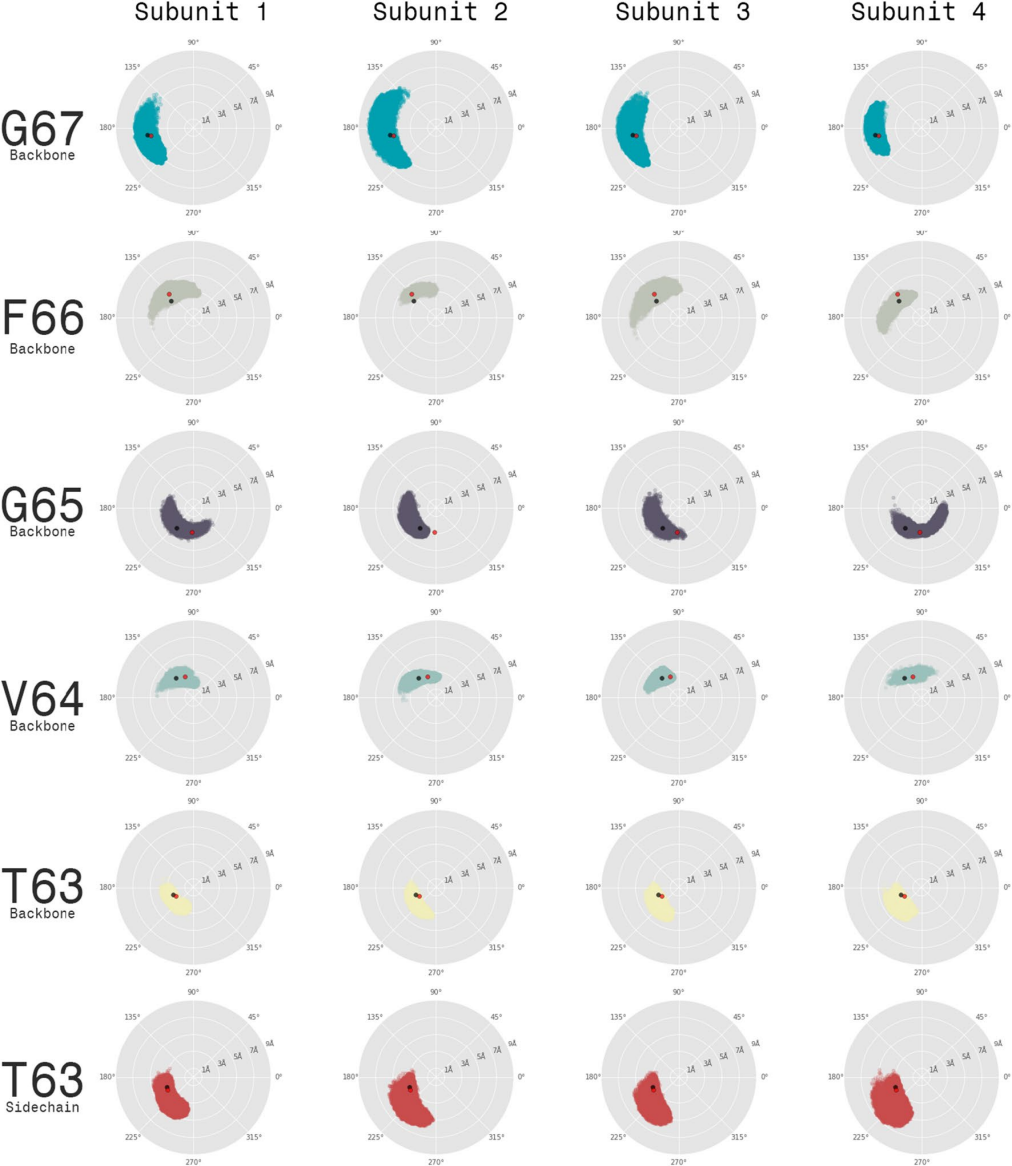
Conformations of the selectivity filter carbonyl groups. The orientation of the T63-G67 carbonyl groups with respect to the conduction pathway was explored. The 2D plots show the $(C, C_\alpha , N, O)$ dihedral and the distance of the carbonyl oxygen to the pore axis. Each point corresponds to a single conformation of ${\hbox {Kcv}}_{\text{PBCV-1}}$ . The rows represent the T63-G67 residues, and the columns—the four channel subunits. On each plot, the two reference points, corresponding to the structures of the NaK2K (red) and hERG channels (black), are shown

Along with the transport of potassium ions, we observed that the backbone of the selectivity filter adopted different conformations during the MD simulations. In particular, the carbonyl groups of the V64-F66 residues fluctuated between the two alternative states, facing either the conduction pathway or the pore helix (Fig. 8). In the latter case, the integrity of the ionic binding sites was disrupted, suggesting an increase of the free energy barrier for passing potassium ions. Indeed in a homologous potassium-selective channel KcsA, such a re-orientation of the protein backbone was shown to affect the ionic transport and was accordingly proposed to be the molecular origin of gating (Bernèche and Roux 2005).

To test whether the conformational change of the selectivity filter is coupled to the ionic transport, for each of the T63-G67 residues, we estimated the $(C, C_\alpha , N, O)$ dihedral and the distance of the carbonyl oxygen to the pore axis (Fig. 8). The density-based clustering performed on these two degrees of freedom revealed distinct conformational ensembles only in the case of V64-F66 . In one of these ensembles, the V64-F66 backbone carbonyls were oriented toward the conduction pathway, and in the other—toward the pore helices. Based on the assignment of each conformation to a specific cluster, we further plotted the orientation of the V64-F66 carbonyl groups along time (Fig. 6a, bottom panel). We noticed that the transition between the two clusters takes place approximately at the same time as the relocation of a potassium ion between adjacent binding sites. For instance, shortly after application of an electric field (within the first few nanoseconds of the simulation), an ion leaves the S2 binding site (Fig. 6a, top panel, first orange trace), an event which coincides with the rotation of two G65 carbonyls from the conduction pathway toward the pore helices (Fig. 6a, bottom panel, cyan, and blue lines). The re-orientation of these carbonyls further takes place when another ion jumps from the S4 to S2 binding sites (Fig. 6a, top panel, blue trace).

Overall, it seems that in ${\hbox {Kcv}}_{\text{PBCV-1}}$ the re-orientation of the V64-F66 residues coincides with the relocation of potassium ions in the selectivity filter, suggesting a mechanism for gating. Since fast gating was previously pinpointed to the selectivity filter, we propose that carbonyl re-orientation constitutes the basis for this process (Abenavoli et al. 2009).

**Fig. 9:**
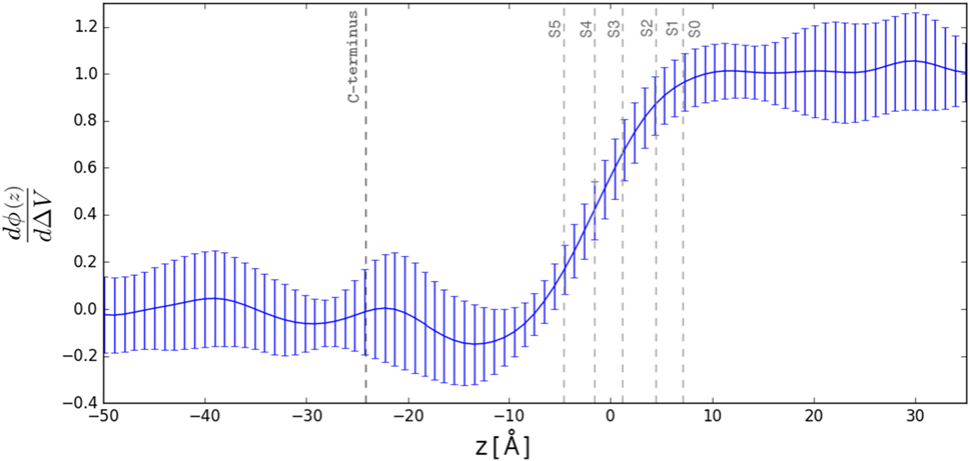
Electrical distance estimated along the ${\hbox {Kcv}}_{\text{PBCV-1}}$ pore axis. A cylinder with a radius of 5 Å centered on the ${\hbox {Kcv}}_{\text{PBCV-1}}$ pore axis was considered for the analysis. The S0–S5 denote the ionic binding sites at the selectivity filter, and the C-termini. The electrical distance was estimated for different ionic configurations of the selectivity filter; the average and the standard deviation are shown

### Charge Transfer at the Selectivity Filter

A process is voltage-sensitive if it is coupled to charge transfer. Here, we have shown that fast gating seems to occur at the selectivity filter. It has previously been suggested that the relocation of ions between the S0–S5 binding sites can be responsible for voltage sensitivity of the selectivity filter gating (Schewe et al. 2016). We hypothesize that a similar mechanism occurs in ${\hbox {Kcv}}_{\text{PBCV-1}}.$

To quantify the charge transfer associated with each ion relocation event, we estimated the electrical distance along the ${\hbox {Kcv}}_{\text{PBCV-1}}$ pore axis (see Methods). The electrical distance reports the reshaping of the electrostatic potential upon application of voltage, and hence can be used to pinpoint the ${\hbox {Kcv}}_{\text{PBCV-1}}$ compartments sensitive to the transmembrane voltage. Figure 9 shows that the electrical distance is approximately constant at the C-termini and in the ${\hbox {Kcv}}_{\text{PBCV-1}}$ cavity, suggesting that none of these compartments participate in charge transfer. In contrast, at the level of the selectivity filter, the electrical distance increases from 0 to 1, indicating that the voltage-sensing events indeed take place in this part of the channel. In particular, an ion jumping from S5 to S4 carries approximately 0.25 e.u., from S4 to S3—0.3 e.u., from S3 to S2—0.25 e.u., from S2 to S1—0.15 e.u., and finally from S1 to the extracellular solution—0.05 e.u. Note that the maximal charge transfer is associated with the relocation events between the S5 and S2 binding sites, i.e., those that are located close to the ${\hbox {Kcv}}_{\text{PBCV-1}}$ cavity.

While here we present a putative charge transfer at the level of the selectivity filter, to quantitatively estimate the charge associated with the fast gating, multiple gating events would need to be observed.

**Fig. 10:**
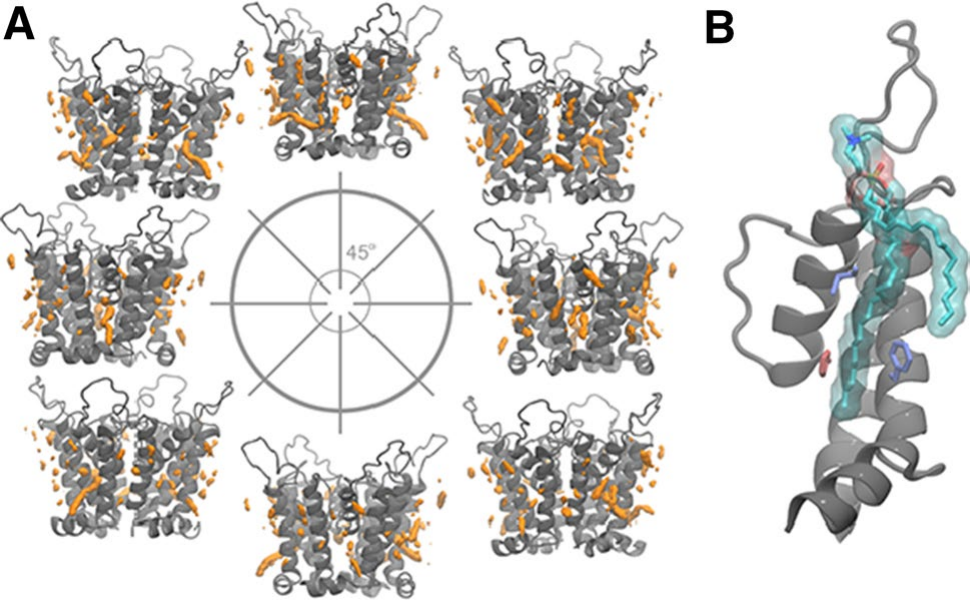
Interactions between ${\hbox {Kcv}}_{\text{PBCV-1}}$ and the lipid bilayer. **a** Average occupancy of the lipids surrounding ${\hbox {Kcv}}_{\text{PBCV-1}}.$ The areas with an occupancy higher than 0.15 are shown in orange. ${\hbox {Kcv}}_{\text{PBCV-1}}$ is colored in gray. Each snapshot shows the channel conformation rotated by $45^o$ with respect to the previous one. **b** A lipid molecule interacting with F19, I54 (blue) and H61 (red)

### ${\hbox {Kcv}}_{\text{PBCV-1}}$ Interactions with the Lipid Bilayer

To characterize the ${\hbox {Kcv}}_{\text{PBCV-1}}$ interactions with the lipid bilayer, we estimated the average occupancy of the lipids surrounding ${\hbox {Kcv}}_{\text{PBCV-1}}$ . Figure 10 shows the areas with the highest average occupancy. Our results suggest that there is a bound lipid in the area where the two adjacent M2 segments contact each other. Interestingly, the tail of this lipid is in contact with F19 and I54 (Figure S7) known to be involved in a network of long-range interactions (Table 2) (Gazzarrini et al. 2004). We hypothesize that the bound lipid mediates the long-range interactions observed experimentally. We also noticed that this lipid interacts with H61, which is evolutionary conserved among viral ion channels but not in ion channels of other organisms. The functional role of H61 is presently unknown; however, our data suggest that it may be involved in the ${\hbox {Kcv}}_{\text{PBCV-1}}$ interactions with the membrane.

## Conclusion

In this contribution, we have proposed a homology model of the ${\hbox {Kcv}}_{\text{PBCV-1}}$ open state, which we have built using a template with a high sequence identity. Overall, we have shown that, first, the ${\hbox {Kcv}}_{\text{PBCV-1}}$ homology model is stable over 369 ns; second, several results of our simulations are in agreement with those obtained previously for homologous potassium-selective channels; and third, the predicted binding affinities for several ${\hbox {Kcv}}_{\text{PBCV-1}}$ blockers correlate with those obtained experimentally. This suggests that our homology model is sufficiently reliable and can be used to explore ${\hbox {Kcv}}_{\text{PBCV-1}}$ function. For instance, using this model, we suggested a molecular basis for fast gating and the origin of its voltage sensitivity. In particular, we propose that fast gating involves re-orientation of selectivity filter carbonyl groups and that its voltage sensitivity can originate from ion transport in the selectivity filter, similarly to what has been reported previously in another $\hbox {K}^+$-selective channel (Schewe et al. 2016). Finally, we have explored ${\hbox {Kcv}}_{\text{PBCV-1}}$ -lipid interactions and found a bound lipid at the interface between adjacent channel subunits. This could help rationalize the results of mutagenesis experiments targeting residues in this region.

## Electronic supplementary material

Below is the link to the electronic supplementary material.

Supplementary material 1 (PDF 3151 KB)
