# Supplementary material for: Exploring the Viral Channel KcvPBCV-1 Function via Computation
Source: J Membr Biol. 2018 Feb 23;251(3):419–30. doi: 10.1007/s00232-018-0022-2 (PMC6028866; doi:10.1007/s00232-018-0022-2)
Supplement: Supplementary file 1 — Supplementary material 1 (PDF 3151 KB) [file 232_2018_22_MOESM1_ESM.pdf]

Supplementary information: Exploring the viral channel Kcv$_{\mathrm{PBCV\text{-}1}}$ function via computation

Alma E. V. Andersson, Marina A. Kasimova, and Lucie Delemotte*

Science for Life Laboratory, Department of Physics, KTH Royal Institute of Technology, Box 1031, SE-171 21 Solna

E-mail: lucie.delemotte@scilifelab.se

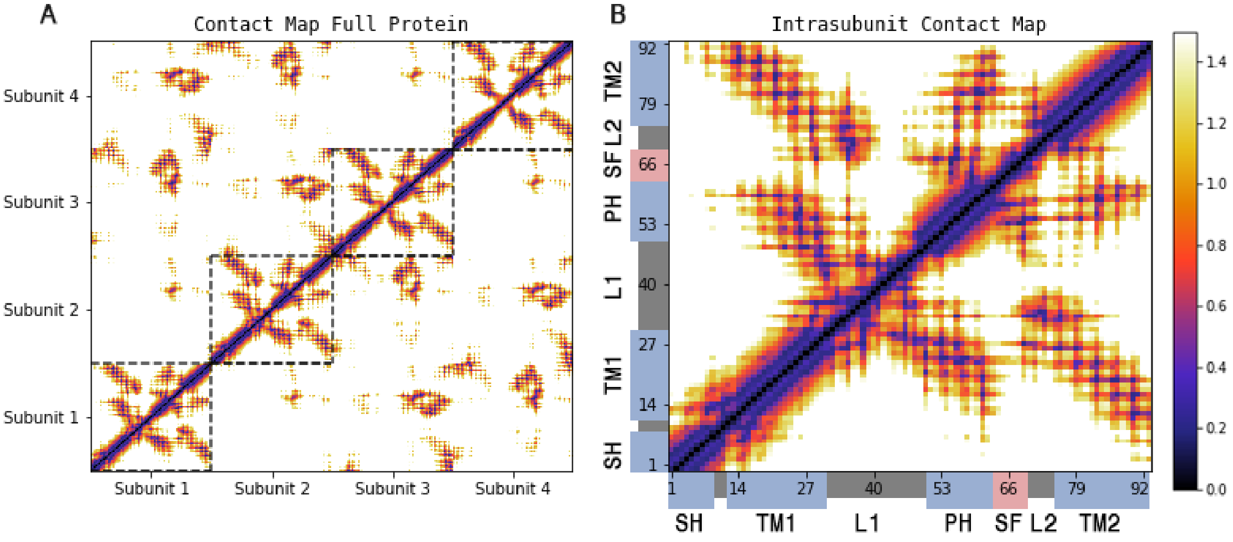

Figure S1: A. Full contact map of average contacts computed from every 1000th frame of the system with no applied external voltage, in nm. B. Close-Up of the intra-subunit interaction seen in A averaged over the four subunits of the channel. Structural features are highlighted in both axes, the labels are given as; SH (Slide Helix), TM1(Transmembrane segment 1), L1 (Loop 1), SF (Selectivity Filter), L2 (Loop2) and TM2 (Transmembrane segment 2).

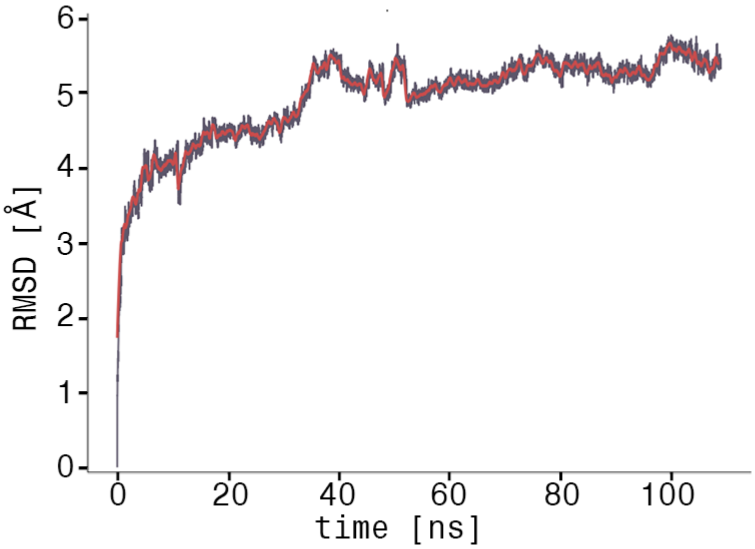

Figure S2: C_α carbon Root Mean Square Displacement (RMSD) as a function of time for the $\sim$ 115 ns trajectory of the Model 0

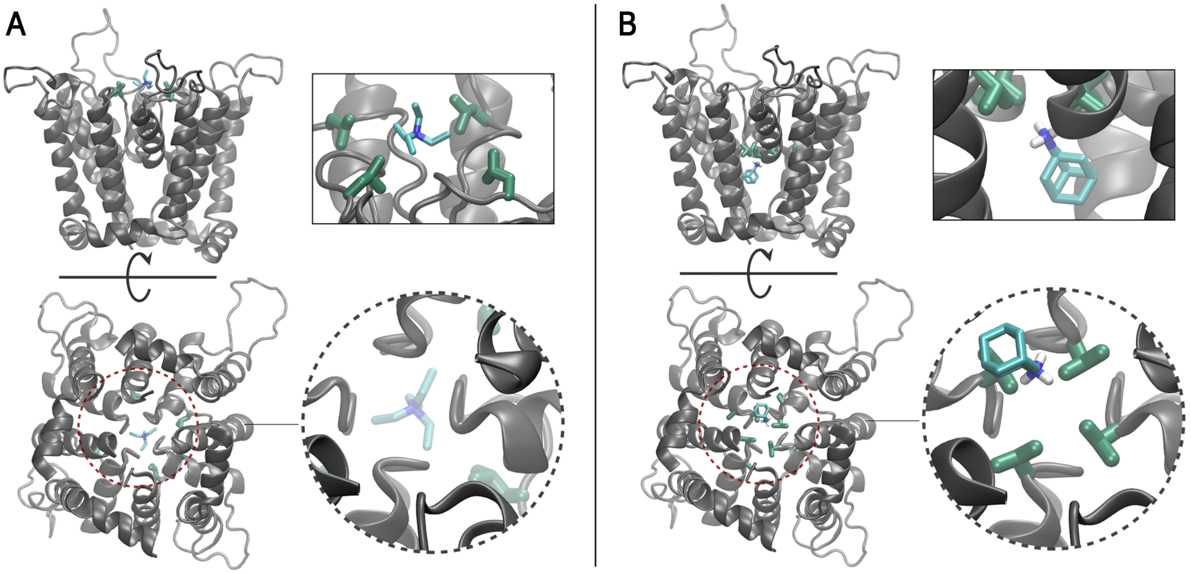

Figure S3: Highest ranked docking poses for TEA (A) and amantadine (B) to the equilibrated model. The side (top panel) and bottom (bottom panel) views are shown. The rectangular and circular insets correspond to the zoomed-in views on the blockers. TEA and amantadine are colored by the atom name: nitrogen - blue, hydrogen - white, and carbon - cyan.

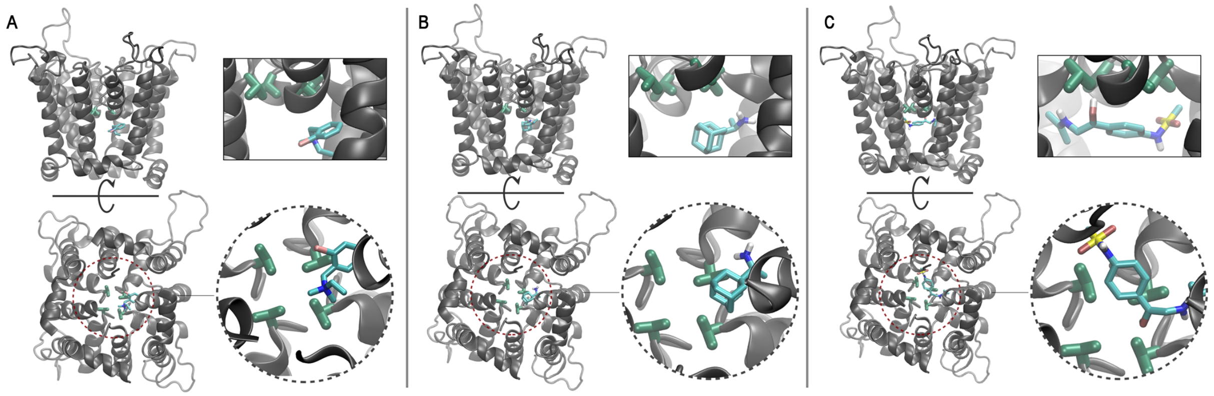

Figure S4: Highest ranked docking poses for bretylium tosylate (A), rimantadine (B) and soltalol (C) to the equilibrated model. The side (top panel) and bottom (bottom panel) views are shown. The rectangular and circular insets correspond to the zoomed-in views on the blockers. TEA and amantadine are colored by the atom name: nitrogen - blue, hydrogen - white, and carbon - cyan.

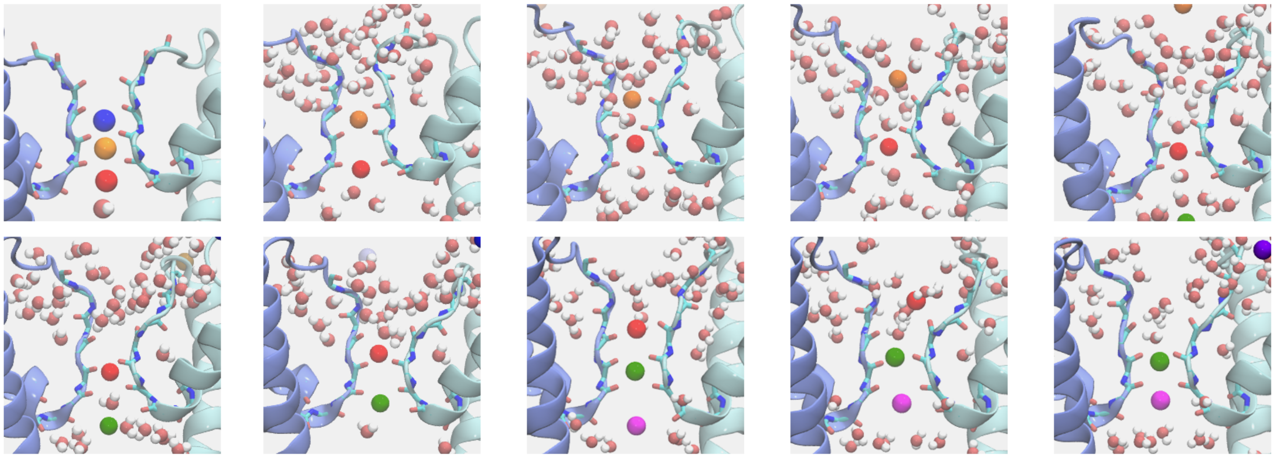

Figure S5: Snapshots taken along conductive events. Time t=0 is set as time for onset of the external transmembrane potential. Each potassium ion is shown in a different color. Shown water molecules are those within 4Å of any of the residues 60 to 68 in the two displayed protein chains.

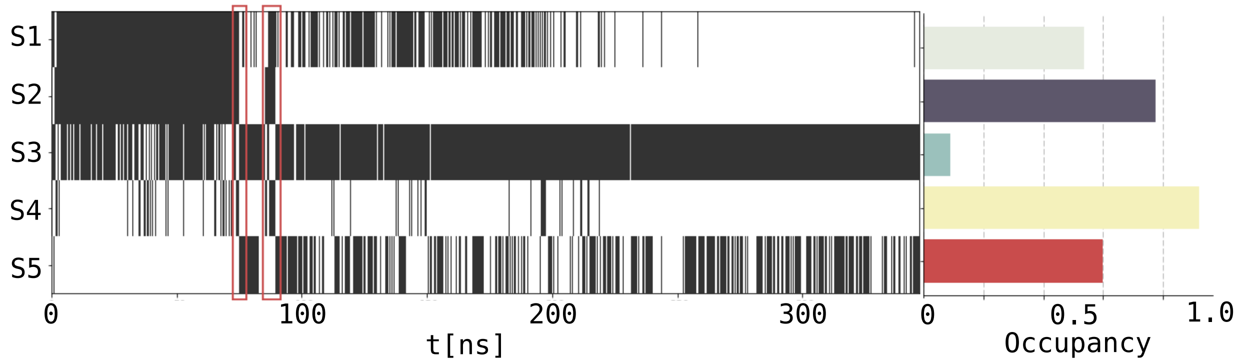

Figure S6: Overview of occupation of all binding sites in the open state model submitted to a +500 mV extrenal transmembrane potential. White and black corresponds to a site being occupied respectively unoccupied, the horizontal bar-graphs indicate the fraction of the time a given site is occupied. Marked in red are the two observed conduction events.

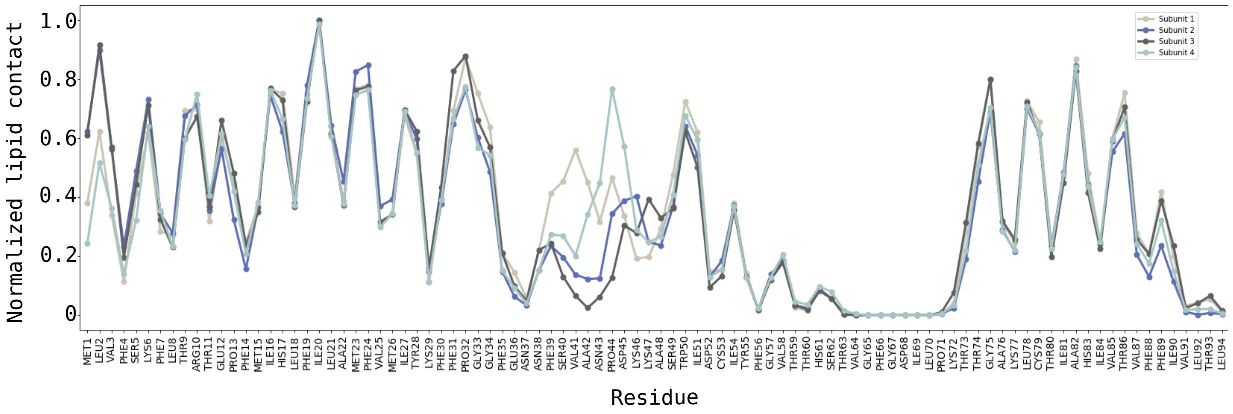

Figure S7: Normalized (by division of maximal value within each subunit) number of atoms belonging to a lipid residue within 1Å of a residue.
